# The Climates and Baths of Great Britain

**Published:** 1902-09

**Authors:** 


**The Climates and Baths of Great Britain.** Vol. II. Pp. xvi., 628. London: Macmillan and Co., Limited. 1902.

This is a continuation of the work undertaken by the Royal Medical and Chirurgical Society of London as long ago as the year 1889. The first volume, published in 1895, was reviewed by us in vol. xiv., p. 361; and the second volume completes and concludes the work accomplished by a committee under the chairmanship of Dr. Theodore Williams. It is on the same lines as the one previously issued, and deals with the central and northern portions of England, with London, and also with Wales and Ireland. The climates and health resorts of the various districts have been considered, and the meteorological data from the different stations compared and contrasted. Special attention has also been given to the diseases which prevail in the several localities, and for the treatment of which the different resorts seem especially suited. The volume contains several maps, of which three are coloured—namely, those of England and Wales and of Ireland, both showing elevations, and also the rainfall map of the British Isles.

These volumes give us a carefully prepared and authoritative account of whatever we may wish to know on the climates and baths of our own country, and they show that there is little need to go abroad in quest of others.

As regards our own locality, we learn that (vol. ii., p. 152)

"In Clifton then, the important residential quarter, the air is stimulating, the rainfall for the West-country not excessive (33.87 inches for the 45 years ending 1897), while humidity is greatly diminished by the configuration of the ground, which allows of admirable natural drainage. It can therefore be truly described as most healthy—an important matter when the number of its schools and scholars is considered."

